# Supplementary material for: Neoadjuvant Metformin Added to Systemic Therapy Decreases the Proliferative Capacity of Residual Breast Cancer
Source: J Clin Med. 2019 Dec 11;8(12):2180. doi: 10.3390/jcm8122180 (PMC6947627; doi:10.3390/jcm8122180)
Supplement: Supplementary file 1 [file jcm-08-02180-s001.pdf]

Neoadjuvant metformin added to systemic therapy reduces the proliferative activity of breast cancer residual disease

SUPPLEMENTARY INFORMATION

Table S1. Association of the interaction between baseline Ki67 score and pCR by treatment arm

	Non-pCR (N, %)	pCR (%)	OR (95% CI)	p-value	OR (95% CI)	p-value
**Ki67 VA**						
<20%	6 (66.67%)	3 (33.33%)	1		1	
≥20%	23 (37.30%)	38 (62.30%)	2.73 (0.39-18.88)	0.309	3.36 (0.76-14.82)	0.110
**Arm**						
B	14 (38.89%)	22 (61.12%)	1		1	
A	15 (44.12%)	19 (55.88%)	0.50 (0.03-8.95)	0.638	0.78 (0.29-2.06)	0.613
**Ki67*Arm**			1.65 (0.08-35.39)	0.749		
**Ki67 ADIA**						
<20%	8 (72.73%)	3 (27.27%)	1		1	
≥20%	21 (35.39%)	38 (64.41%)	5.73 (0.53-61.75)	0.150	4.76 (1.13-20.09)	0.034
**Arm**						
B	14 (38.89%)	22 (61.12%)	1		1	
A	15 (44.12%)	19 (55.88%)	1.20 (0.07-19.63)	0.898	0.93 (0.34-2.51)	0.879
**Ki67*Arm**			0.74 (0.04-14.79)	0.845		

Table S2. Association of the interaction between baseline Ki67 score and pCR in the metformin-containing arm A

	Non-pCR (N, %)	pCR (%)	OR (95% CI)	p-value
**Ki67 VA**				
<20%	3 (75.00%)	1 (25.00%)	1	
≥20%	12 (40.00%)	18 (60.00%)	4.50 (0.42-48.53)	0.215
**Ki67 ADIA**				
<20%	5 (71.43%)	2 (28.57%)	1	
≥20%	10 (37.04%)	17 (62.96%)	4.25 (0.69-26.13)	0.118

Table S3. Association of the interaction between baseline Ki67 score and pCR in the control arm B

	Non-pCR (N, %)	pCR (%)	OR (95% CI)	p-value
**Ki67 VA**				
<20%	3 (60.00%)	2 (40.00%)	1	
≥20%	11 (35.48%)	20 (64.52%)	2.73 (0.39-18.88)	0.309
**Ki67 ADIA**				
<20%	3 (75.00%)	1 (25.00%)	1	
≥20%	11 (34.38%)	21 (65.63%)	5.73 (0.53-61.75)	0.150

Table S4. Association of the interaction between baseline Ki67 score and pCR by treatment arm adjusted by HR status

	Non-pCR (N, %)	pCR (%)	OR (95% CI)	p-value	OR (95% CI)	p-value
**Ki67 VA**						
<20%	6 (66.67%)	3 (33.33%)	1		1	
≥20%	23 (37.30%)	38 (62.30%)	2.86 (0.40-20.25)	0.293	3.25 (0.72-14.60)	0.125
**Arm**						
B	14 (38.89%)	22 (61.12%)	1		1	
A	15 (44.12%)	19 (55.88%)	0.54 (0.03-9.97)	0.680	0.71 (0.26-1.93)	0.503
**Ki67*Arm**			1.36 (0.06-30.51)	0.845		
HR						
ER and/or PR+	*19 (47.50%)*	*21 (52.50%)*	*1*		*1*	
ER and PR-	*10 (27.78%)*	*20 (66.67%)*	*1.81 (0.65-5.02)*	*0.253*	*1.83 (0.67-5.05)*	*0.242*
**Ki67 ADIA**						
<20%	8 (72.73%)	3 (27.27%)	1		1	
≥20%	21 (35.39%)	38 (64.41%)	6.98 (0.61-80.01)	0.119	5.57 (1.26-24.74)	0.024
**Arm**						
B	14 (38.89%)	22 (61.12%)	1		1	
A	15 (44.12%)	19 (55.88%)	1.14 (0.07-19.96)	0.929	0.83 (0.30-2.11)	0.723
**Ki67*Arm**			0.70 (0.03-14.56)	0.815		
HR						
ER and/or PR+	*19 (47.50%)*	*21 (52.50%)*	*1*		*1*	
ER and PR-	*10 (33.33%)*	*20 (66.67%)*	*2.28 (0.78-6.63)*	*0.131*	*2.21 (0.77-6.35)*	*0.140*

Table S5. Association of the interaction between baseline Ki67 score and pCR in the metformin-containing arm a adjusted by HR status

	Non-pCR (N, %)	pCR (%)	OR (95% CI)	p-value
**Ki67 VA**				
<20%	3 (75.00%)	1 (25.00%)	1	
≥20%	12 (40.00%)	18 (60.00%)	3.90 (0.35-43.51)	0.269
HR				
ER and/or PR+	*9 (52.94%)*	*8 (47.06%)*	*1*	
ER and PR-	*6 (35.29%)*	*11 (64.71%)*	*1.81 (0.03-7.46)*	*0.410*
**Ki67 ADIA**				
<20%	5 (71.43%)	2 (28.57%)	1	
≥20%	10 (37.04%)	17 (62.96%)	4.94 (0.75-32.62)	0.097
HR				
ER and/or PR+	*11 (57.89%)*	*8 (42.11%)*	*1*	
ER and PR-	*6 (31.58%)*	*13 (68.42%)*	*2.45 (0.56-10.69)*	*0.233*

Table S6. Association of the interaction between baseline Ki67 score and pCR in the control arm B adjusted by HR status

	Non-pCR (N, %)	pCR (%)	OR (95% CI)	p-value
**Ki67 VA**				
<20%	3 (60.00%)	2 (40.00%)	1	
≥20%	11 (35.48%)	20 (64.52%)	2.86 (0.40-20.33)	0.294
HR				
ER and/or PR+	*10 (43.48%)*	*13 (56.52%)*	*1*	
ER and PR-	*4 (30.77%)*	*9 (69.237%)*	*1.81 (0.42-7.87)*	*0.428*
**Ki67 ADIA**				
<20%	3 (75.00%)	1 (25.00%)	1	
≥20%	11 (34.38%)	21 (65.63%)	6.80 (0.58-79.14)	0.126
HR				
ER and/or PR+	*10 (41.67%)*	*14 (58.33%)*	*1*	
ER and PR-	*4 (23.53%)*	*13 (76.47%)*	*2.10 (0.45-9.84)*	*0.347*
